# Single-cell sequencing technologies in bladder cancer research: Applications and challenges

**DOI:** 10.3389/fgene.2022.1027909

**Published:** 2022-10-19

**Authors:** Tianqi Lyu, Yuanbin Lin, Kerong Wu, Zhanglei Cao, Qian Zhang, Jianping Zheng

**Affiliations:** ^1^ Cixi Institute of Biomedical Engineering, Ningbo Institute of Materials Technology and Engineering, Chinese Academy of Science (CAS), Ningbo, China; ^2^ Department of Urology, Ningbo First Hospital, School of Medicine Ningbo University, Zhejiang University Ningbo Hospital, Ningbo, China

**Keywords:** single-cell sequencing, bladder cancer, heterogeneity, biomarkers, precision medicine

## Abstract

Bladder cancer is among the most common malignant tumors with highly heterogeneous molecular characteristics. Despite advancements of the available therapeutic options, several bladder cancer patients exhibit unsatisfactory clinical outcomes. The lack of specific biomarkers for effective targeted therapy or immunotherapy remains a major obstacle in treating bladder cancer. The rapid development of single-cell techniques is transforming our understanding of the intra-tumoral heterogeneity, thereby providing us with a powerful high-throughput sequencing tool that can reveal tumorigenesis, progression, and invasion in bladder tumors. In this review, we summarise and discuss how single-cell sequencing technologies have been applied in bladder cancer research, to advance our collective knowledge on the heterogeneity of bladder tumor cells, as well as to provide new insights into the complex ecosystem of the tumor microenvironment. The application of single-cell approaches also uncovers the therapeutic resistance mechanism in bladder cancer and facilitates the detection of urinary-exfoliated tumor cells. Moreover, benefiting from the powerful technical advantages of single-cell techniques, several key therapeutic targets and prognostic models of bladder cancer have been identified. It is hoped that this paper can provide novel insights into the precision medicine of bladder cancer.

## Introduction

Bladder cancer is the most common malignant tumor that affects the urinary system and is associated with high morbidity and mortality (Antoni et al., 2017). Several risk factors such as sex, age, lifestyle, pathogenic microbial infections, and drugs may augment the bladder cancer risk (Lin et al., 2006; Letašiová et al., 2012; Burger et al., 2013; Berdik, 2017). Despite advancements of the available therapeutic approaches, the clinical outcomes of bladder cancer patients remain unsatisfactory, and the corresponding 5-year survival rate remains almost unchanged. Bladder cancer is one of the highly heterogeneous human cancers, often involving frequent genomic alterations and molecular subtype diversity, and it may be the first cause of therapeutic failure; therefore, the elucidation of the molecular and cellular heterogeneity of bladder cancer is necessary (Genitsch et al., 2019). Probably, an in-depth understanding of the unique molecular characteristics and cellular heterogeneity involved in the initiation, development, and pathogenesis of bladder cancer can improve the clinical treatment and prognosis, as well as contribute to the promotion of its diagnosis and treatment towards precision medicine.

Over the past few decades, with the rapid development of next-generation sequencing and molecular biology technologies, research on the biological characteristics and genotyping of bladder cancer has made considerable strides (Pietzak et al., 2017; Roy et al., 2017; Wu et al., 2019; Li et al., 2021a; Tran et al., 2021). Bladder cancer can be categorised into three subtypes based on its histological features: urothelial carcinoma, squamous cell carcinoma, and adenocarcinoma. Of these subtypes, urothelial tumors account for approximately 90% of cases, while squamous and glandular-type tumors are rare (Kantor et al., 1988; Knowles and Hurst, 2015). The 2016 World Health Organisation grading system has stratified bladder cancer into non-muscle-invasive bladder cancer (NMIBC) and muscle-invasive bladder cancer (MIBC) (Compérat et al., 2019). At the first clinical diagnosis, approximately 85% of bladder cancer patients are diagnosed with NMIBC, and up to 80% of these patients have at least one recurrence, whereas disease progression into MIBC occurs in approximately 30% of these patients (Batista et al., 2020; van den Bosch and Alfred Witjes, 2011). It is difficult for doctors to predict the risk of recurrence and progression of NMIBC based on the conventional classification system.

As of today, enormous efforts are being undertaken to outline the diverse molecular subtypes and collect crucial biological information on bladder cancer. Several researchers have proposed that the molecular and genetic heterogeneities of bladder cancer cells are critical to diagnosis and treatment of the disease (Thomsen et al., 2017; Audenet et al., 2018; Sjödahl et al., 2019; Minoli et al., 2020). However, despite these advances, clinical therapies based on the molecular subtypes and genetic features of bladder cancer often show several limitations such as poor efficacy, drug resistance, and recurrence due to tumor heterogeneity (Casadei et al., 2019; Kamoun et al., 2019; Zhu et al., 2020). In general, the molecular mechanisms for the initiation and development of bladder cancer remain elusive, and the current molecular subtyping systems do not depict the cellular heterogeneity of bladder carcinoma. In the era of precision medicine, we should consider the vital role played by inter-tumoral and intra-tumoral heterogeneities. Decoding the heterogeneity of bladder cancer cells at the genetic and cellular levels, refinement of the molecular types, and discovering new ways to overcome resistance are the main issues that should be addressed in the studies on bladder cancer.

**FIGURE 1:**
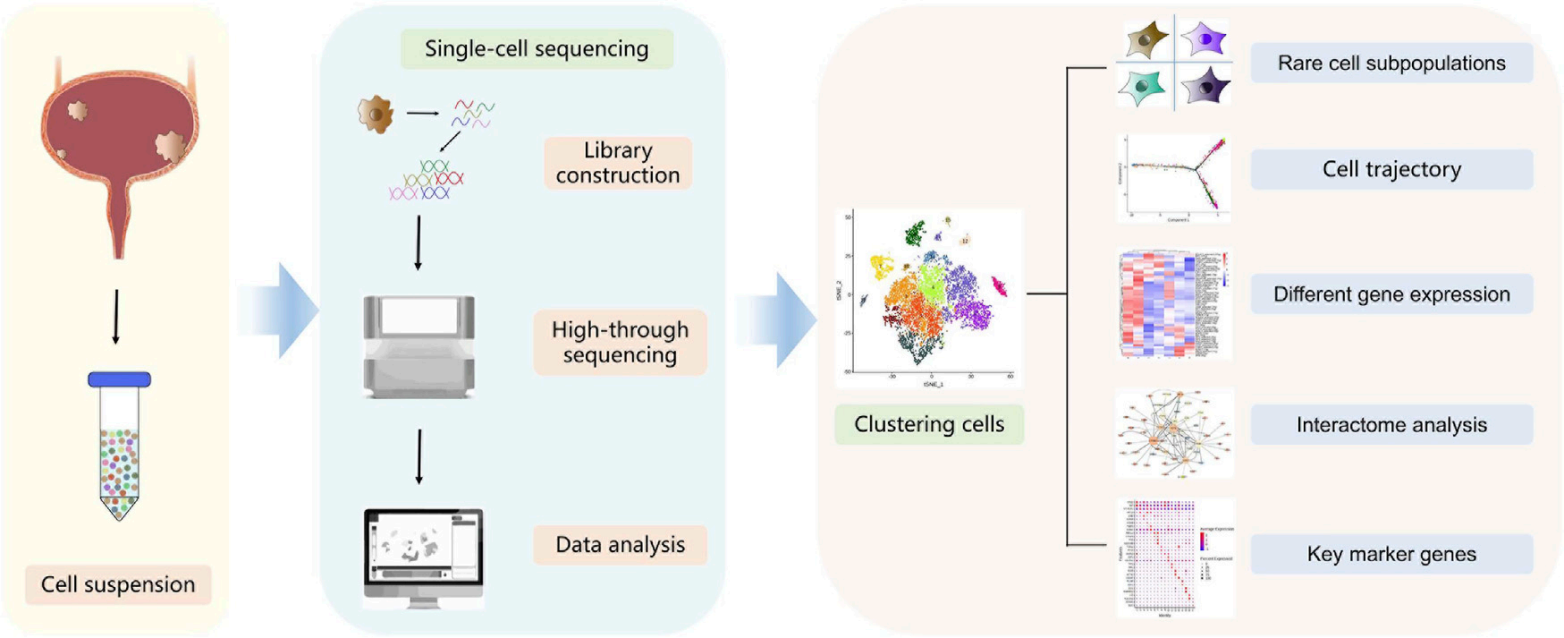
Schematic diagram of the single-cell sequencing workflow. Cells are dissolved from bladder tumors into single-cell suspension. Single cells are then processed into the reaction system followed by reverse transcription, amplification, library construction and sequencing. Sequencing data are processed *via* bioinformatic analysis such as cell clustering, cell trajectory, different gene expression, interactome or key marker genes analysis.

In 2009, Tang et al. reported the single-cell RNA sequencing (scRNA-seq) technology for the first time, which helped solve the problem of cell heterogeneity that was difficult to be solved by bulk RNA sequencing (Tang et al., 2009). The rapid developments in single-cell techniques allow us to interrogate the transcriptional, genomic, proteomic, epigenomic, metabolic, and multi-omic characteristics of thousands of individual cells (Kashima et al., 2020) (Table 1). This powerful high-resolution and high-throughput sequencing tool has revolutionised our understanding of cancer biology and tumor cytology (Stuart and Satija, 2019; Xing et al., 2020). These technologies have helped in successfully deciphering the compositional complexity and clonal heterogeneity in tumors (Lei et al., 2021). Indeed, single-cell techniques have multiple applications in cancer research, which include the identification of dynamic gene expression profiles during tumor progression, discovery of novel cell subpopulations, determination of cell status and phenotype switches, analysis of the regulatory pathways of key genes, and identification of potential therapeutic targets (Papalexi and Satija, 2018) (Figure 1). Owing to the excellent technical advantages, single-cell techniques have undoubtedly promoted the precision and accuracy of molecular cancer research to new levels. The past decade has seen unprecedented advances in bladder cancer studies, which can be ascribed mainly to single-cell techniques (Table 2). In this review, we summarise pioneering research in the field and discuss the latest progress in the aspects such as cellular heterogeneity, tumor microenvironment (TME), drug resistance, and prognosis of bladder cancer. All of these further inspire and foster research on clinically relevant diagnosis, prognosis prediction, targeted therapy, and personalised therapeutic strategies for bladder cancer.

**TABLE 1:** Single-cell omics technologies.

Omics	Methods	Feature
Single-cell transcriptome	STRT-seq, Smart-seq, CEL-seq, MARS-seq, Drop-seq, 10×Genomics, Seq-Well, Microwell-seq, SCRB-seq, SPLit-seq	mRNA in single cells was analyzed for gene expression quantification, functional enrichment, and metabolic pathways
Single-cell genome	MDA, DOP-PACR, MALBAC, LIANTI, META-CS	Analysis of single nucleotide site variation, CNV, and single-cell genomic structure variation
Single-cell epigenome	scBS-seq, scRRBS, scAba-seq, scATAC-seq, Drop-ChIP, scChIC-seq, CUT&Tag, Single-cell Hi-C	Analysis of DNA methylation to resolve epigenetic mechanisms
Single-cell protein mass spectrometry	SCoPE-MS, SCoPE2, sc-CyTOF	Conduct in-depth proteomic studies of key cell subsets that have been targeted, and further study of mechanisms at the protein level
Single-cell immune repertoire	scTCR-seq, scBCR-seq	Analyze the interaction status of T cells and B cells with tumor cells to study the immune response mechanism

**TABLE 2:** Summary of the studies in bladder cancer using single-cell technologies.

Events	Species	Sample/Tissue	Technology	References
The heterogeneity and differentiation of bladder urothelial cells	Human, mouse	Normal bladder urothelium, injured bladder mucous, organoids	scRNA-seq	(Santos et al., 2019; Yu et al., 2019; Li et al., 2021b; Cheng et al., 2021)
The heterogeneity of bladder cancer	Human, mouse	Bladder tumors, adjacent normal mucosae	scRNA-seq, scATAC -seq	(Zhang et al., 2016; Sfakianos et al., 2020; Wang et al., 2021a; Kerzeli et al., 2021; Lai et al., 2021)
The genetic characteristics of bladder cancer stem cells	Human	Bladder tumor and normal adjacent tissue	scWES-seq, scDNA-seq, scATAC-seq	(Li et al., 2012; Yang et al., 2017; Wang et al., 2021a)
The composition of the bladder tumor microenvironment	Human, mouse	Bladder tumors and normal adjacent tissue, young and aged murine bladders	scRNA-seq, scTCR-seq, sc-CyTOF	(Chen et al., 2020; Ligon et al., 2020; Oh et al., 2020; Wang et al., 2021b; Feng et al., 2021)
The detection of urinary exfoliated tumor cells	Human	Urinary exfoliated tumor cells	scWGS-seq	(Chen et al., 2018; Wang et al., 2020)
The drug resistance in bladder cancer	Human, mouse	Human primary bladder tumor cells, PDX human cells, and PDX mouse TME cells	scRNA-seq	(Tanaka et al., 2018; Lee et al., 2020; Wang et al., 2021c)
The prognosis of bladder cancer	Human	Bladder tumors and normal adjacent tissue	scRNA-seq, snRNA-seq	(Gouin et al., 2021; Lai et al., 2021; Zhou et al., 2021)

## Uncovering the heterogeneity of bladder urothelial cells through single-cell sequencing

**FIGURE 2:**
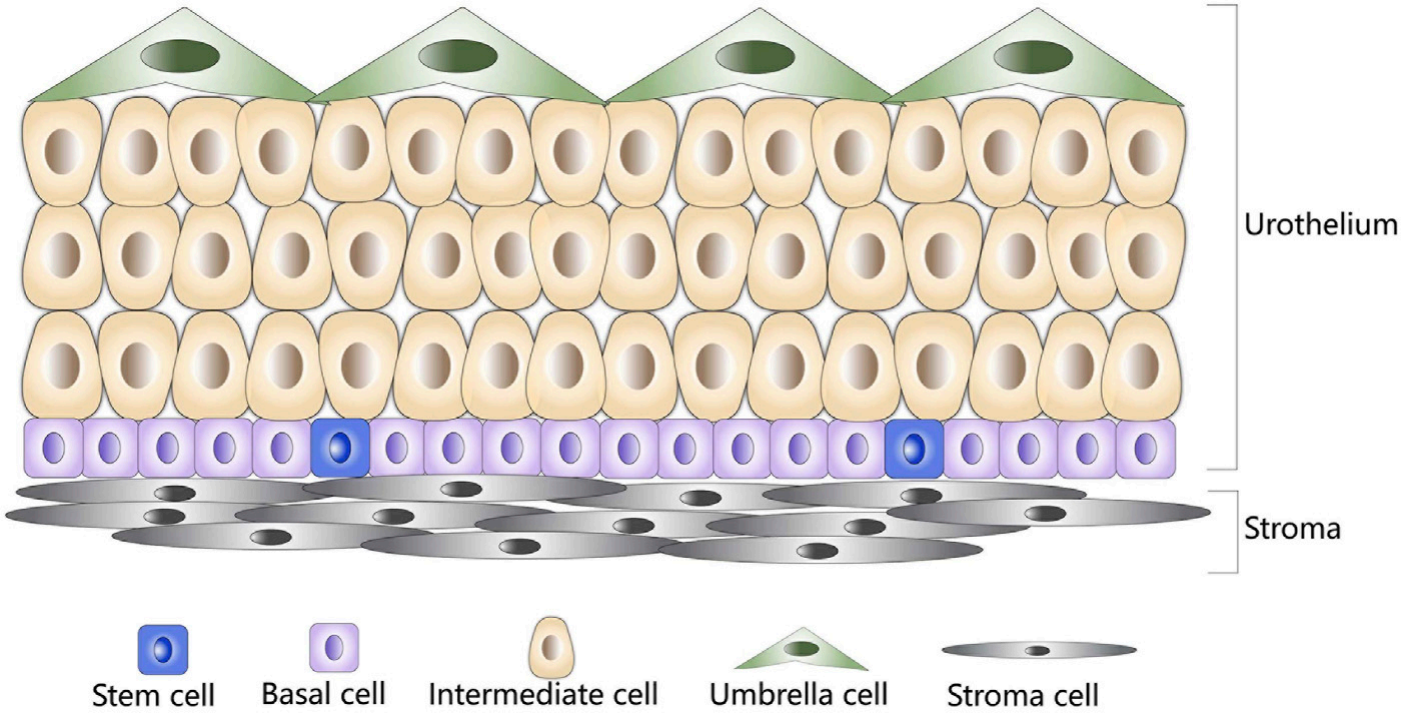
Schematic diagram of normal bladder urothelium. Stem cells are shown in blue, basal cells are shown in purple, intermediate cells are shown in brown, umbrella cells are shown in green, and stroma cells are shown in gray.

The urothelium of the urinary bladder is a particular hierarchically organised tissue that serves as the strongest urine–blood barrier in the body. The adult urothelium can be histologically classified as the transitional epithelium consisting of three types of cells, namely basal, intermediate, and umbrella cells (Figure 2). Among these cells, the basal cells are small and polygonal and form a single layer that directly contacts the basement membrane. Intermediate cells are pyriform and form multiple cell layers. Umbrella cells are large binuclear or multinuclear cubic cells that form a single layer in direct contact with the urinary space (Apodaca, 2004; Khandelwal et al., 2009; Ho et al., 2012). Usually, the bladder urothelium regenerates rapidly upon injury, which depends on the proliferation of urothelial stem cells (Ho et al., 2012). Accumulating evidence suggests that minor subpopulations of basal cells, characterised by the expressions of keratin 5 (KRT5) and keratin 14 (KRT14), possess self-renewal capacity and give rise to all cell types of the urothelium during natural and injury-induced regeneration processes, which represents the cellular origins of urothelial carcinoma (Shin et al., 2011; Papafotiou et al., 2016). Reported research demonstrates that a vast majority of tumor lesions of the bladder arise from the urothelial cells (Castillo-Martin et al., 2010). Therefore, elucidation of the primitive cellular differentiation status, stem cell differentiation, and genetic alteration characteristics of bladder urothelial may provide critical biological insights for understanding the molecular mechanism of associated bladder diseases.

To gain an insight into the cell subtypes and urothelial differentiation characteristics, Li et al. performed scRNA-seq to detect urothelial cells digested and isolated from the mouse bladder urothelium (Li et al., 2021b). The authors discovered that the ASPM-labelled basal-like cells might be the progenitor cells of the other subpopulations that could participate in regeneration of the bladder urothelium. In particular, a novel superficial-like cell population (Plxna4^+^ urothelial cells) may play a priming role in the initiation of resisting inflammation and repairing of the responses of the bladder urothelium. Yu et al. compared the single-cell transcriptomic maps of human and mouse bladders to determine both the conservative and heterogeneous aspects of human and mouse bladder evolution (Yu et al., 2019). The authors identified two new types of human bladder cells: ADRA2A^+^ and HRH2^+^ interstitial cells and TNNT1^+^ epithelial cells. Of these, ADRA2A^+^ interstitial cells appear to play a special role in the human bladder, while TNNT1^+^ epithelial cells are also present in rat and mouse bladder tissues. Developmental trajectory analysis revealed that the bladder epithelial cells could be transformed from basal cells to intermediate cells and then into umbrella cells or TNNT1^+^ epithelial cells. Another study recapitulated the differentiation and function of urothelial cells by establishing mouse urothelial organoids, wherein the authors identified five cell clusters by utilising scRNA-seq and reported that the CD49f/CD44 high-labelled urothelial cell subpopulation exhibits the highest organoid-forming potential (Santos et al., 2019; Yu et al., 2019). In previous studies, CD49f and CD44 have been proposed as markers for aggressive basal/squamous bladder cancer subtypes (Lerner et al., 2016; Sjödahl et al., 2017). Notably, the investigation unveiled the essential role of Notch signalling in the differentiation of normal urothelial cells, which was elusive in the past bulk organoid transcriptomics studies. Furthermore, the study confirmed that gene mutations in the Notch pathway could induce urothelial tumors.

Recently, Cheng et al. explored the cell heterogeneity involved in the repair and regeneration processes of bladder urothelial cells and focused on the crosstalk between cell subsets (Cheng et al., 2021). When compared with the normal urothelial cells, two cycling cell populations (i.e., basal cells and luminal cells) were highly enriched under injured conditions. A study demonstrated that urothelium regeneration is mediated by distinct division patterns of the cycling basal and intermediate cells (Wang et al., 2018); in this study, the single-cell analysis confirmed that this discrepancy of cell cycle progression might be attributable to the difference in cell adhesion (Cheng et al., 2021). By analysing the lineage relationship among urothelial cell subpopulations, the authors found that cycling basal cells and cycling intermediate cells represent two different states of the urothelium. Notably, cycling intermediate cells could act as the direct progenitors of superficial cells, where the latter was directly repaired by the former, which verifies the previous hypothesis. According to cell–cell communication analyses, Acta2^+^Cd34^+^ myofibroblasts have the most significant influence on the proliferation of urothelial cells, and both Bmp and Fgfr signaling are involved in this process (Cheng et al., 2021). These results together supported the view that epithelial–myofibroblast crosstalk plays a key role in urothelial homeostasis and repair.

In summary, these results further reinforce the conclusion that bladder urothelial cells are highly heterogeneous and provide fundamental insights into the correlation between diverse cell phenotypes of the bladder urothelium and bladder diseases.

## Single-cell sequencing in the cellular heterogeneity of bladder cancer

Discerning the heterogeneity of individual tumor cells carries clinical implications for predicting prognosis and treatment responses of bladder cancer. For instance, in the murine and human bladder cancer model systems, single-cell transcriptome analyses revealed the presence of multi-lineage gene expression phenotypes. In addition, among the epithelial cell clusters, cell subpopulations with concomitantly pronounced expressions of basal, luminal, and EMT claudin-related genes were characterised, and the cellular expression of these lineage markers was found to be dynamic either during tumor progression or in response to the treatment. Analysis of transplantable MIBC models demonstrated that these specific multi-lineage subtypes could define the cancer cells capable of tumor formation and lineage plasticity, which could be distinguished by the surface antigen CD49f in conjunction with the epithelial markers (Sfakianos et al., 2020). Finally, investigators affirmed the exact composition of the intra-tumoral cells derived from multi-lineage subpopulations as showing inherent heterogeneity and lineage plasticity of the epithelium. Data from a recent study revealed the heterogeneity of epithelium in bladder cancer patients with different pathological tumor stages (Lai et al., 2021). According to previous reports, bladder cancer is a urinary tract epithelium-derived cancer, and epithelial–mesenchymal transition (EMT) promotes the transition from NMIBC to MIBC (Cao et al., 2020). The malignant cell-specific gene expression suggested that basal-like cells were invasive precursors that displayed EMT characteristics in NMIBC. Recently, Wang et al. elucidated the spectrum of EMT associated with bladder cancer subtypes and identified that TCF7 promotes EMT in corroboration with single-cell ATAC sequencing (scATAC-seq) (Wang et al., 2021a). This evidence suggested that the epithelial cells represent the malignant cells of bladder cancer and that the upregulation of EMT is essential for driving local bladder tumor cells’ invasion and progression.

To further investigate the tumor heterogeneity involved in the transition from NMIBC to MIBC, Kerzeli et al. established a novel urothelial carcinoma model of Hgf-Cdk4R24C mice and identified eight heterogeneous cell clusters of urinary epithelium origin by utilising single-cell transcriptomic analyses (Kerzeli et al., 2021). Unlike the urothelial cells in healthy bladders, there were urothelial cell clusters with highly proliferative or cancer stemness gene expression characteristics in carcinogens-induced bladder tumors. Notably, these cells displaying cell cycle dysregulation gene expression profiles were considered asaggressive subclones. These results are in line with the reports of a study that observed intra-tumoral heterogeneity of human urothelial bladder cancer, identifying it as clonal mutations according to their genetic characteristics (Heide et al., 2019). Since the histopathological feature of Hgf-Cdk4R24C bladder tumors was urothelial carcinoma with squamous differentiation (UC/SCC), the proposed model would be a helpful tool for determining the tumor biological characteristics and the treatment response of UC/SCC.

Histologically, different from UC/SCC, tumors in bladder cancer patients with pure squamous cell components are defined as squamous cell carcinoma of the bladder (SCCB). Although SCCB accounts for <10% of primary bladder cancer, it shows frequent recurrence and metastasis when compared with urothelial carcinoma, which highlights the more complex intra-tumoral heterogeneity of SCCB (Minato et al., 2018; Lin et al., 2019). In 2016, a study conducted scRNA-seq on the tumor-normal paired tissues from an SCCB patient and revealed intra-tumoral heterogeneity and the potential mechanisms of SCCB (Zhang et al., 2016). The authors considered that genes with different expression patterns particularly enriched in the MAPK signaling pathway were involved in tumor evolution and heterogeneity formation. Especially, a keratinocyte-specific POU transcription factor, POU2F3, was associated with squamous epithelial stratification, indicating that POU2F3 may be a crucial biomarker of SCCB.

In general, these findings reveal the complex intra-tumoral and inter-tumor heterogeneities of bladder cancer, which can explain its diverse molecular and clinical phenotypes. These identified aggressive subclones and biomarkers may help enhance our understanding of the progression of bladder cancer, thereby facilitating individualised diagnosis and treatment.

## Single-cell sequencing for investigating the genetic characteristics of bladder CSCs

Cancer stem cells (CSCs) possess the capability of self-renewal and are responsible for the initiation and development of tumors. Past studies have suggested that bladder CSCs possess high tumorigenicity, drug resistance, metastasis, and typical biomarkers (Ho et al., 2012). As early as 2012, Li et al. employed the single-cell exome sequencing technology to analyse the genetic landscape of bladder carcinoma and deciphered the mechanism of its occurrence and development (Li et al., 2012). Their study suggested that bladder cancer cells are derived from a single ancestral cell that formed two distinct tumor cell subgroups in the subsequent evolutionary process. Furthermore, they demonstrated that the ancestral bladder cancer cells had a monoclonal phenotype with multiple mutation driver gene candidates, among which *ATM*, *COL6A3*, and *KIAA1958* were discovered as novel subclone-specific genes. These candidate cancer-related genes could drive the initiation of carcinogenesis and the development of subsequent cell lineages involved in cancer progression, which best matches the clonal evolution model. These findings explained that the genesis of bladder cancer was multi-gene mutation drive and multi-factorial, which in turn provided evidence for deciphering how tumors evolve into difficult-to-treat metastases. However, there remains an ambiguity related to whether ancestral cells inferred by the authors represent the initial tumor cells (Heng et al., 2006; Li et al., 2012; Ren et al., 2018; Zhang and Zhang, 2020).

To further investigate the genetic basis and origin of bladder CSCs, Yang et al. conducted single-cell sequencing on 59 cells from three human bladder cancer specimens and examined their phylogenetic status (Yang et al., 2017). Their research demonstrated that bladder CSCs originated from bladder epithelial stem cells or bladder cancer non-stem cells, thus providing genetic evidence to support the hypothesis of CSC origin. Moreover, 21 key gene mutations in bladder CSCs were discovered, which involved the genes related to cell cycle regulation, transcription regulation, chromatin remodeling, cell differentiation, and self-renewal. These specific mutations are critical to the acquisition of bladder CSC stemness. Moreover, the alterations of the three genes *ARID1A*, *GPRC5A*, and *MLL2* play a crucial role in conferring stemness to the bladder cancer non-stem cells, as confirmed by co-mutation gene assays. Wang and others performed single-cell ATAC sequencing and found that bladder CSCs were enriched during tumor recurrence with elevated expression of EZH2. The specific molecular mechanism is that EZH2 maintains H3K27me3-mediated repression of the *NCAM1* gene, thereby inactivating cellular invasiveness and stemness transcriptional programs (Wang et al., 2021a).

Overall, several potential key genes related to the characteristics of bladder CSCs have been uncovered so far. Nevertheless, further studies on the function and clinical applications are warranted to determine whether these candidate genes can act as bladder cancer biomarkers to guide targeted therapy.

## Single-cell sequencing reveals the complexity of the bladder TME

The tumorigenesis, progression, and invasion of cancer cells are closely related to their surrounding microenvironment (Bryan, 2015; Song et al., 2019). Notably, bladder cancer is one of the least immune invasive cancers (Chen et al., 2017), and its complex TME may account for the poor response to immunotherapy. The composition of the bladder cancer TME was poorly understood. Recently, Chen et al. utilised the single-cell transcriptome sequencing technology to produce a cell atlas of the entire TME of bladder cancer (Chen et al., 2020). The authors found that bladder tumor cells express low levels of MHC-II molecules, suggesting that the downregulated immunogenicity may contribute to the avoidance of immune detection. Furthermore, the LAMP3^+^ dendritic cell subgroup expressed various cytokines, such as CCL17, CCL19, and CCL22, contributing to the formation of an immunosuppressive TME. Most importantly, two types of cancer-associated fibroblasts (CAFs) were identified. Among these fibroblasts, inflammatory CAFs (iCAFs) might promote the proliferation of bladder tumor cells and stromal cells and then recruit immune cells to the tumor region. Therefore, iCAFs were considered as the critical factor for the progression of bladder cancer and targeting CAFs might be an optimal choice for bladder cancer treatment in the future. Feng et al. integrated mass cytometry and imaging mass cytometry to investigate the MIBC TME at the single-cell proteomic level (Feng et al., 2021). They identified a specific CSC cluster (ALDH^+^ PD-L1^+^ ER-β^−^) that is associated with poor prognosis, improving the general understanding of the complexity of the TME in bladder cancer. Recently, Wang et al. also used single-cell mass cytometry to compare the characteristics of the TME between two groups MIBC patients (Wang et al., 2021b). They found that there was an immunosuppressive microenvironment in the immune-low group, with higher expression of PD-1 and Tim-3 on Tregs, and a higher proportion of PD-1^+^ Tregs.

Adaptive immune responses throughout the body are mainly coordinated by T lymphocytes. The phenotype of infiltrating T lymphocytes in the TME largely determines the response to immunotherapy (Hatogai and Sweis, 2020). Baras et al. for instance, indicated that the ratio of CD8 to Treg tumor-infiltrating lymphocyte density in the pre-treatment tissues can predict the response of bladder cancer to the platinum-based neoadjuvant chemotherapy (Baras et al., 2016). Notably, the current research on immunotherapy mainly focuses on the cytotoxic CD8^+^ T-cell-mediated response. Although the presence of cytotoxic CD4^+^ T cells has been validated in non-small cell lung cancer and hepatocellular carcinoma (Zheng et al., 2017; Guo et al., 2018), the extent of their heterogeneity and contribution to immunotherapy remains unclear. Through scRNA-seq and paired TCR-seq, Oh et al. analysed the tumor-infiltrating lymphocyte heterogeneity of bladder cancer (Oh et al., 2020). Notably, cytotoxic CD4^+^ T cells, rather than typical CD8^+^ T cells, were significantly enriched in bladder tumors. Cytotoxic CD4^+^ T cells recognised MHC-II antigens to kill the bladder tumor cells and lyse autologous tumor cells in a way that is inhibited by autologous Treg. Of note, signatures of these cytotoxic CD4^+^ T cells were significantly correlated with the clinical response to anti-PD-L1 therapy in bladder inflammation samples. Collectively, these findings have breakthrough research significance in the immunotherapy strategy of bladder cancer. In future treatments, the balance between cytotoxic CD4^+^ and regulatory T-cell status can be manipulated to provide therapeutic benefits to bladder cancer patients.

Aging can also lead to immune environment changes of the bladder, which induces corresponding bladder diseases (Maserejian et al., 2013). However, how and why bladder diseases become more prevalent with aging remains obscure. Single-cell transcriptomics analysis from the aged mouse bladders revealed the composition of the bladder immune cell repertoire, including novel subpopulations of macrophages and dendritic cells and notable changes in the immune repertoire. When compared with the young bladder tissues, T and B cells were highly enriched in aged bladders that constituted the organised bladder tertiary lymphoid tissues (bTLTs), which was supported by histological analyses (Ligon et al., 2020). Lymphoid aggregates have also been reported in some bladder tumor models, albeit they remain poorly characterised (Pitzalis et al., 2014). This report highlights that local immune environment dysfunction inside the bladder may be a potential mechanism that drives age-related bladder diseases. Moreover, the study provides an insight into the link among aging, inflammation, and bladder diseases.

In summary, the aforementioned single-cell studies have demonstrated the cell composition in the bladder TME and determined the molecular characteristics of immune cell subpopulations. Nevertheless, research in this field is still in its infancy.

## Single-cell sequencing for studying urinary-exfoliated tumor cells of bladder cancer

Liquid biopsy for the detection of urinary-exfoliated tumor cells (UETCs) provides a hope for non-invasive screening and precision medicine for bladder cancer treatment. Before the development of bladder metastasis lesions, mutant tumor cells are released from the primary site of the bladder lesion or metastatic site to the patients’ urine. UETCs carry original information on each stage of the bladder cancer progression and have hence been utilised as potential biomarkers for urinary cytology detection (Critelli et al., 2016). However, UETCs are extremely rare and easily confounded by non-tumor cells, and the current related detection methods suffer from limited specificity or sensitivity (Yafi et al., 2015; Huang et al., 2021). To accurately and reliably identify UETCs, Chen et al. developed a novel microfluidic immunoassay method to separate and collect intact UETCs (Chen et al., 2018). This method combined the microfluidic immunoassay technology with two oncoproteins, namely CK20 and CD44v6, with frequent overexpression in the urothelial layers to achieve highly sensitive marking and separation of UETCs. Later, single-cell whole-genome sequencing and copy number variation (CNV) analysis were performed on 12 captured UETCs to achieve an accurate diagnosis of bladder cancer (with a specificity of 71.5%). To further improve the sensitivity of UETC detection and overcome the low-throughput limitation of single-cell DNA sequencing, Wang et al. performed single-cell CNV profile analysis combined with a cellular marker hexokinase 2 (HK2) to detect a total of 385 UETCs from eight urothelial carcinoma patients (Wang et al., 2020). Past studies have shown that, as a key enzyme in glucose metabolism, HK2 was significantly overexpressed in various types of cancers (Xu et al., 2018). Therefore, HK2 constitutes a promising biomarker for detecting urothelial cancer by identifying high glycolysis and metabolic abnormalities in UETCs. The study of the CNV profiles or oncogenic driver mutation signatures of bladder cancer, when combined with an HK2 threshold, achieved a specificity of >90% for UETC detection (Wang et al., 2020).

Overall, urine-based cellular or metabolic markers, when combined with single-cell sequencing, demonstrated superior sensitivity to the conventional urine cytology detection, which may enable high-sensitivity detection of UETCs in patients’ urine at the early and symptom-free stages.

## Research on the therapeutic resistance of bladder cancer with single-cell sequencing

For non-metastatic bladder cancer patients, neoadjuvant cisplatin-based chemotherapy before radical cystectomy is the first-line of standard clinical treatment (Witjes et al., 2020). However, treatment failure due to drug resistance is common in bladder cancer, and approximately 40% of the patients show recurrence and metastasis after cystectomy (Kamoun et al., 2019). The second-line treatments for cisplatin-resistant and metastatic bladder cancer are the immune checkpoint inhibitors involving specific oncogene targeting agents, and approximately 20% of the patients exhibit a positive response to the treatment (Hargadon et al., 2018). Presently, the mechanisms of resistance to systemic therapy of bladder cancer are largely unknown, and molecular biomarkers for selecting effective second-line treatments are still lacking.

The emergence of drug resistance has been closely associated with intra-tumoral heterogeneity profiles. However, whether it results from pre-existing rare clones or new genome mutations remains controversial (Saunders et al., 2012). To investigate the mechanism of bladder cancer chemotherapy resistance, Tanaka et al. provided a single-cell atlas of bladder cancer treated with platinum-based chemotherapy and revealed the intra-tumoral heterogeneity states before and after treatment (Tanaka et al., 2018). By establishing an acquired platinum-resistant subline of urinary urothelial cancer cell line 5,637, the authors determined that 12 genes were considered as candidate platinum-resistance genes. Further evaluation of their clinical relevance demonstrated that only COX7B was associated with high mortality, while low levels of COX7B predicted a poor prognosis. Notably, the surrogate marker CD63 could distinguish low-COX7B subclones; thus, the authors speculated that COX7B and CD63 together contribute to platinum resistance in platinum-naïve bladder cancer.

The complex interactions between the TME and tumor cell-intrinsic mechanisms contribute to therapeutic failure and tumor evolution (Junttila and de Sauvage, 2013). To analyse tumor progression and TME changes during the clinical course of bladder cancer, Lee et al. depicted the tumor single-cell landscape of HRAS mutations in chemo-resistant metastatic MIBC (Lee et al., 2020). Clinically, the use of tipifarnib for advanced MIBC with HRAS mutations showed a significant therapeutic effect but was unable to achieve a complete response (Appels et al., 2005; Wang et al., 2017). Consequently, there is an urgent need to monitor the evolutionary trajectories of tumor cells and the surrounding TME during the clinical treatment course of MIBC. With the help of scRNA-seq and PDX models, a unique cell subpopulation with inherent resistance to tipifarnib was proven to exist at the time of tumor pre-treatment (Lee et al., 2020). Most importantly, the tumor subtypes with HRAS mutations show high expression levels of PD-L1, which indicates that alterations in tumor cells and TME by tipifarnib can be reversed by PD-1/PD-L1 immune checkpoint inhibitors. Therefore, the tipifarnib combined immunotherapy strategy may overcome the treatment failure and show favorable clinical responses.

Although immunotherapy offers great potential, not all MIBC patients can benefit from the current therapeutic regimens; in fact, improper treatment can increase mortality (Kamoun et al., 2019). For gaining in-depth understanding of the resistance mechanism of anti-PD-1/PD-L1 therapies, Wang et al. analysed the gene signatures of metastatic urothelial carcinoma resistance to PD-1/PD-L1 blockade through bulk transcriptome sequencing data (Wang et al., 2021c). Later, the cellular characteristics of the MIBC specimens were scrutinised through scRNA-seq. The results obtained suggested that the associated cells had diverse heterogeneity and that myeloid phagocyte could significantly upregulate the pro-tumorigenic inflammatory factors and downregulate the antigen-presenting genes. Hence, the authors anticipated that the PD-1/PD-L1 resistance of urothelial carcinoma was related to the balance of adaptive immunity and pro-tumorigenic inflammation in an individual TME.

In general, the occurrence of drug resistance in bladder cancer may be attributed to the pre-existing rare clones. Although the current findings are insufficient to explain the fundamental reasons for the treatment failure of bladder cancer, these studies can still provide novel insights into the drug resistance mechanism.

## Single-cell sequencing in the prognosis of bladder cancer

A reliable prognostic prediction is essential to guide effective clinical treatment, which can safely avoid treatment failure and drug resistance (Wu et al., 2020). Zhou et al. collected the scRNA-seq profiles of 2075 cells from a bladder cancer patient using the GEO database and identified 14 different cell subgroups (Zhou et al., 2021). Then, with combined co-expression analyses and MATH value, 96 genes related to intra-tumoral heterogeneity, including several novel genes (such as *KDELR3*, *GPSM3*, *RFC4*, *RPA3*, *IFI27L2*, and *APH1A*) that may be correlated with bladder cancer progression, were screened. Moreover, a heterogeneity-related score model that could predict clinical treatment was established. This study revealed that the risk model based on scRNA-seq and intra-tumoral heterogeneity could help identify novel biomarkers and predict the clinical treatment outcomes, thereby providing new clues for understanding the intra-tumoral heterogeneity mechanisms of bladder cancer. Recently, Gouin III et al. using single-nucleus RNA-sequencing demonstrated that a novel epithelial cell phenotype marked by high expression of CDH12, which predicting poor prognosis for neoadjuvant chemotherapy (Gouin et al., 2021). In contrast, CDH12-enriched cells exhibit superior response to immune checkpoint therapy. CDH12-enriched cells highly express ligands for CD49a as well as PD-L1 and PD-L2, and co-localize with exhausted T-cells, providing one explanation for immune checkpoint blockade efficacy in these tumors.

The interaction between tumor cells and other cells in the TME has been widely studied (Wang et al., 2007); however, little attention has been paid to the communication among malignant cells. To analyse the guidance of cell communication between tumor cells on the prognosis, Lai et al. implemented single-cell analysis to study the ligand–receptor interaction in bladder cancer malignant cells (Lai et al., 2021). The authors discovered that six ligand genes and eight receptor genes were associated with the basal subtype tumors, while the high expression of the HBEGF-EGFR ligand–receptor pair predicted a poor prognosis. In addition, luminal subtype-related genes included three ligand genes and seven receptor genes, while the high expression of the FAM3B-LRP5 ligand–receptor pair indicated a more favorable outcome. These results highlight the relevance of cellular communication between tumor cells in the prognosis of bladder cancer for the first time.

Altogether, predictive models and biomarkers associated with the prognosis of bladder cancer have been identified so far. However, further prospective, large-scale, and multi-center clinical trials are warranted to validate their clinical utility.

## Conclusion and perspectives

**FIGURE 3:**
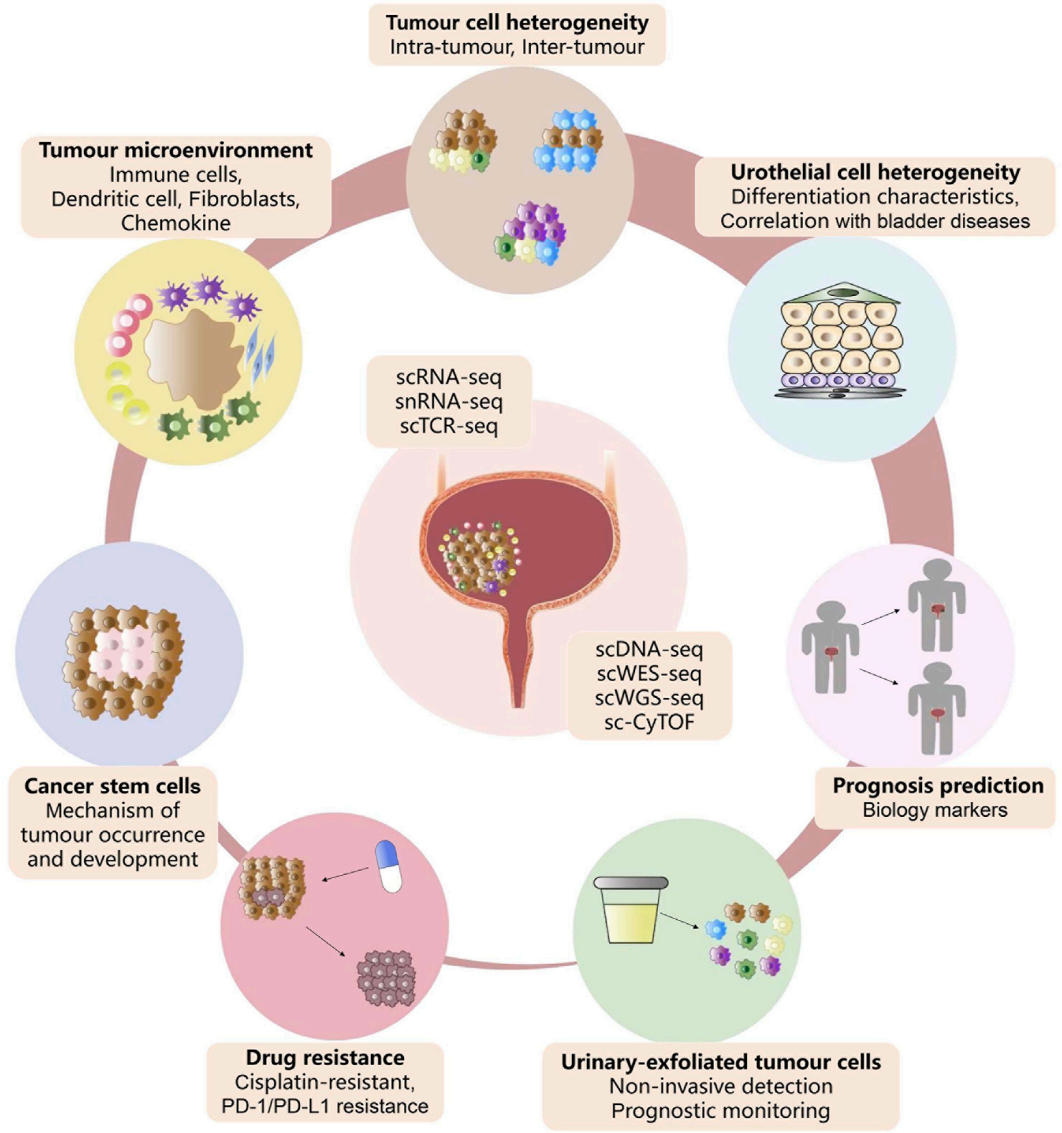
Advances of single-cell sequencing in bladder cancer. Summary of the current application of single-cell sequencing technology in the cell heterogeneity, tumor microenvironment, drug resistance, cancer stem cells, urinary-exfoliated tumor cells, and prognosis of bladder cancer.

As a powerful high-throughput sequencing tool, single-cell techniques enable interrogating homeostatic and pathogenic cell populations with an exceptionally high resolution, thereby driving bladder cancer biomedical research to a new level of precision. These innovative methods have been extensively utilised to identify or interrogate the heterogeneity of bladder urothelial cells and bladder tumor cells, TME signatures, therapy resistance, UETCs, and CSCs (Figure 3). The application of scRNA-seq has provided a wide range of transcriptional expression profiles, which are essential for discovering rare cell subpopulations, discerning intra-tumor heterogeneity, monitoring the dynamic progression of bladder tumors, and revealing the drug resistance mechanisms. More importantly, these results of scRNA-seq could help identify novel carcinogenic driver biomarkers that may serve as therapeutic targets or prognostic factors ultimately guiding clinical therapies. In addition, single-cell DNA sequencing coupled with urine-based liquid biopsy has been found valuable in non-invasive screening for bladder cancer and monitoring the therapeutic efficacy. Overall, single-cell sequencing technologies have revolutionised our understanding of bladder tumor cytology, tumor biology, and tumor immunology, as they offer broader and deeper insights into prognosis prediction and treatment strategies to inspire future drug discovery.

The past several years have witnessed unpredictable advances in single-cell sequencing technologies in the cancer field (Papalexi and Satija, 2018; Liu et al., 2021). However, the novel applications of single-cell technologies in characterising bladder cancer currently remain insufficient when compared with other human malignancies (Zang et al., 2021; Wei et al., 2022). First, existing single-cell sequencing studies have been limited to providing transcriptome or genomic information for a few specific types of bladder cancer patients, especially those lacking proteomic and cellular spatial information (Chen et al., 2018; Chen et al., 2020; Wang et al., 2020). Second, given the difficulty in obtaining the bladder urothelium of healthy individuals, single-cell sequencing studies on the link between the urothelium and bladder disease are limited to mice (Maserejian et al., 2013; Santos et al., 2019; Li et al., 2021b; Cheng et al., 2021). Third, drug resistance and metastasis often occur in bladder cancer patients, relevant single-cell research should not be limited to bladder cancer cell lines and PDX models (Tanaka et al., 2018; Lee et al., 2020). It remains indispensable to expand the study on the mechanism of bladder cancer drug resistance and metastasis through single-cell techniques in the future. As a result, our present understanding of bladder cancer cellular matrix components, TME, and drug resistance mechanisms are far from adequate. Therefore, it is a long and arduous process to comprehensively understand the underlying cellular or molecular mechanisms of bladder tumors, and plentiful studies need to be conducted.

Besides, due to the complex and expensive processes, single-cell sequencing could not be applied to profile large cohorts of tumor samples (Zhang et al., 2016; Chen et al., 2018; Chen et al., 2020; Oh et al., 2020; Wang et al., 2020). Even in real-world clinics, single-cell sequencing results cannot be applied to individualized therapy due to the rapidly deteriorating health status of patients (Lee et al., 2020). Notably, limited by the algorithm, it is difficult to identify accurate proportion of rare cell subsets in batch sequencing data (Li et al., 2012; Yang et al., 2017; Chen et al., 2020). This remains a big issue in bioinformatics analysis of single-cell sequencing data, and more proofs are needed to validate these single-cell analysis results. With the ongoing technological developments, single-cell sequencing is expected to become more high-throughput, with a low cost involved, simplified data analysis process, and reduced time expenditure, that can provide better accuracy and sensitivity to collectively promote the larger-scale single-cell sequencing efforts in bladder cancer and accelerate its clinical application. Furthermore, advanced techniques such as the application of single-cell multi-omics approaches and spatial transcriptomics will help us comprehensively reveal the pathogenesis and drug resistance mechanisms of bladder cancer. With the deepening of our understanding of the cellular dynamics of bladder tumor cells, the diagnosis and treatment of bladder cancer are expected to usher in a new era of precision medicine, improve the efficacy of personalised medicine, and ultimately save the lives of bladder cancer patients.
